# Porcine Xenograft and Epidermal Fully Synthetic Skin Substitutes in the Treatment of Partial-Thickness Burns: A Literature Review

**DOI:** 10.3390/medicina57050432

**Published:** 2021-04-30

**Authors:** Herbert L. Haller, Sigrid E. Blome-Eberwein, Ludwik K. Branski, Joshua S. Carson, Roselle E. Crombie, William L. Hickerson, Lars Peter Kamolz, Booker T. King, Sebastian P. Nischwitz, Daniel Popp, Jeffrey W. Shupp, Steven E. Wolf

**Affiliations:** 1 HLMedConsult, Zehetlandweg 7, 4060 Leonding, Austria; 2 Lehigh Valley Health Network 1200 S. Cedar Crest Blvd. Kasych 3000, Allentown, PA 18103, USA; sibloeb@yahoo.com (S.E.B.-E.); swolf@utmb.edu (S.E.W.); 3 Department of Surgery—Burn Surgery, The University of Texas Medical Branch and Shriners Hospitals for Children, 301 University BLVD, Galveston, TX 77555, USA; lubransk@UTMB.EDU; 4 Department of Surgery, UF Health Shands Burn Center, University of Florida, 1600 SW Archer Rd, Gainesville, FL 32610, USA; Joshua.Carson@surgery.ufl.edu; 5 Connecticut Burn Center, Yale New Haven Heal System, 267 Grant St, Bridgeport, CT 06610, USA; Roselle.Crombie@bpthosp.org; 6 Memphis Medical Center Burn Center, 890 Madison Avenue, Suite TG032, Memphis, TN 38103, USA; whicker1@uthsc.edu; 7 Division of Plastic, Aesthetic and Reconstructive Surgery, Department of Surgery, Medical University, 8053 Graz, Austria; lars.kamolz@medunigraz.at (L.P.K.); sebastian.nischwitz@medunigraz.at (S.P.N.); daniel.popp@medunigraz.at (D.P.); 8 Division of Burn Surgery, Department of Surgery, 101 Manning Drive CB #7206, Chapel Hill, NC 27599, USA; bookert@email.unc.com; 9 The Burn Center, Department of Surgery, MedStar Washington Hospital Center, 110 Irving St NW, Washington, DC 20010, USA; Jeffrey.W.Shupp@medstar.net

**Keywords:** dressing changes, epidermal skin substitute, grafting, healing time, infection rate, partial thickness burns, porcine xenograft, resorbable, suprathel, synthetic, workload

## Abstract

*Background and Objectives*: Porcine xenografts have been used successfully in partial thickness burn treatment for many years. Their disappearance from the market led to the search for effective and efficient alternatives. In this article, we examine the synthetic epidermal skin substitute Suprathel^®^ as a substitute in the treatment of partial thickness burns. *Materials and Methods*: A systematic review following the PRISMA guidelines has been performed. Sixteen Suprathel^®^ and 12 porcine xenograft studies could be included. Advantages and disadvantages between the treatments and the studies’ primary endpoints have been investigated qualitatively and quantitatively. *Results*: Although Suprathel had a nearly six times larger TBSA in their studies (*p* < 0.001), it showed a significantly lower necessity for skin grafts (*p* < 0.001), and we found a significantly lower infection rate (*p* < 0.001) than in Porcine Xenografts. Nonetheless, no significant differences in the healing time (*p* = 0.67) and the number of dressing changes until complete wound healing (*p* = 0.139) could be found. Both products reduced pain to various degrees with the impression of a better performance of Suprathel^®^ on a qualitative level. Porcine xenograft was not recommended for donor sites or coverage of sheet-transplanted keratinocytes, while Suprathel^®^ was used successfully in both indications. *Conclusion*: The investigated parameters indicate that Suprathel^®^ to be an effective replacement for porcine xenografts with even lower subsequent treatment rates. Suprathel^®^ appears to be usable in an extended range of indications compared to porcine xenograft. Data heterogeneity limited conclusions from the results.

## 1. Introduction

Contemporary burn care aims at rapid closure of open wounds, either temporarily or permanently. Wound closure reduces infectious complications and downregulates inflammation and other detrimental systemic responses. Moreover, it curbs the hypermetabolic response and supports re-establishment of undisturbed energy expenditure in the mitochondria [1,2].

Porcine xenograft (PX) (Mölnlyke, Peachtree Corners, GA, USA) and biosynthetic and synthetic dressings, such as human skin allografts, amniotic membrane, Biobrane^®^ (Dow Hickman/Bertek Pharmaceuticals, Sugarland, TX, USA), Dermagraft™ (Organogenesis, Canton, MA, USA), Appligraf^®^ (Organogenesis, Canton, MA, USA), OrCel^®^ (ORTEC int. Inc., New York, NY, USA), Hyalomatrix^®^ (Medline Industries, Northfield, IL, USA), Transcyte^®^ (Takeda Pharmaceutical Co. Ltd., Tokyo, Japan), and Suprathel^®^ (ST) (Polymedics Innovations GmbH, Denkendorf, Germany) as epidermal skin substitutes, have been used for the closure of partial-thickness wounds. The requirements of these products include safety, ease of application, a short healing time, effectiveness, hypo- allergenicity, and non-oncogenicity, while being able to be stored easily and cost-effective. The PX EZ Derm^®^ was used with numerous indications but is not available on the market anymore, yielding the need for finding the optimal replacement and delivering the motivation for this review.

This paper compares the biological pig skin-derived skin substitute (EZ Derm) to a fully synthetic and biodegradable epidermal substitute (ST) based on the published literature. After describing general product characteristics, we conducted a modified systematic review of the literature to evaluate the suitability or advantages of products other than PX. 

## 2. Materials and Methods

Given the absence of studies directly comparing PX and ST^®^ treatment in burns, we extracted data from studies comparing either PX or ST^®^ to other treatment modalities.

### 2.1. Data Retrieval

PubMed^®^, Science Direct^®^, and Google Scholar^®^ were searched. The primary strategy was to find studies describing the results of the different products in partial thickness burns.

### 2.2. Study Selection

Studies were selected according to the PRISMA guidelines. We selected articles published in peer-reviewed journals or reviewed and published abstracts of an international meeting on burns.

### 2.3. Exclusions

Studies on the treatment of mainly or exclusively deep partial-thickness burns were not described. We excluded studies on donor site areas, porcine small intestine submucosa, genetically modified pigskin, and full-thickness burns. We excluded in vitro studies and studies that were not relevant, mentioning one treatment method without numerical data. Non-English articles or articles without full-text have been excluded as well.

### 2.4. Search Method and Search Results Based on the PRISMA Flow Chart

**Figure 1:**
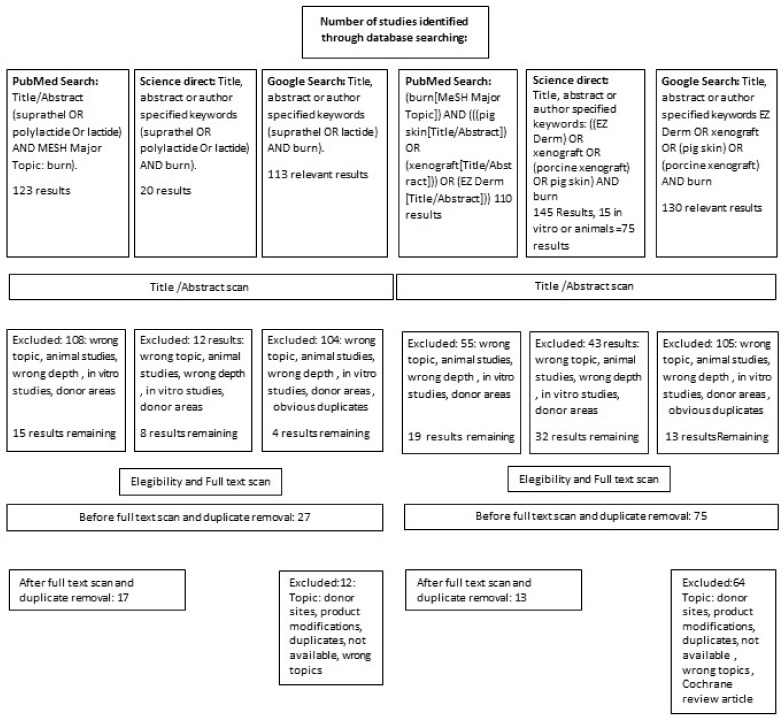
The PRISMA procedure.

Figure 1 shows the Prisma procedure.

The following data were retrieved from the studies: study type (prospective, retrospective, randomized, non-randomized, descriptive); study population (pediatric, adult, or mixed); sex distribution (male, female); age; cause of burn (scald, flame, contact, flash); timing of epidermal substitute application; description of use in donor sites (Yes/No); information on detailed burn depth (partial superficial, partial deep, or full-thickness burn); technique of dressing application; wound ground preparation; dressing method and dressing change frequency; healing time; information and percentage of infections; hypertrophic scarring percentage; product replacement frequency and necessity; hospital length of stay (LOS).

### 2.5. Statistics

In many of the primary studies, the variance was not described. The validity of these studies’ statistical output is limited and can only be seen as an approximation. Only studies themselves could have been compared and not individuals treated in the studies. The data were weighted on the number of patients in the studies. Medians were transformed to means as described by Hozo et al. [1] when indicated for comparison. SPSS 20 was used for statistics. The Kolmogorov–Smirnov test was used to identify data for normal distribution and the Levene test for homogeneity of variance. Student *T*-Test was used for normally distributed data and Kruskal–Wallis and Welch’s test for not normally distributed ones. Being well aware of the shortcomings, the statistical efficiency was calculated on pooled data from the studies [2]. A level of *p* < 0.05 was considered statistically significant.

The number of average dressing changes was calculated by dividing healing time by interval of dressing changes in the studies.

### 2.6. Effect Size of Treatment Modalities

The effect size calculated can only be a rough estimate due to the heterogeneity of studies. The standard effect size was calculated using the SPSS *T*-Test and Two-Sample *T*-Test Calculator from statistics Kingdom for unknown unequal standard deviation [3]. The effect size interpretation was made with no effect when d_Cohen_ was <0.1, a small effect with a d_Cohen_ of 0.2 to 0.4, a medium effect with d_Cohen_ of 0.4–0.6, and a large effect d_Cohen_ of >0.6.

## 3. Results

In total, 29 studies have been found with two of them describing pediatric and adults separately and where counted separately. There was no special evaluation of mixed populations (pediatric and adult). After exclusion of non-relevant studies (see above), 17 and 16 studies have been included dealing with ST and PX, respectively.

### 3.1. Quality of Studies

In the ST studies, nine out of 17 studies were done prospectively. Five of the studies were randomized.

In the PX studies, six out of 16 studies were prospective and four of them were randomized. Details are given in Table 1 and Table 2.

### 3.2. Inclusion Criteria

Studies showed various inclusion criteria, burn causes, time to admission, total burn surface area (TBSA), TBSA grafted, and data quality. For some topics, data reports were sparse, and therefore these topics are not discussed further.

### 3.3. Biocompatibility and Systemic Effects

Wound closure with PX reduces pain, fluid, and heat loss [20,21,22]. Gal and non-Gal antigens are essential pig xenoantigens, causing an endothelial complement-mediated injury, resulting in PX thrombosis [23] which will not be incorporated. A “xenograft reaction” is described anechoically but not published yet by users with an increased leukocytosis and elevated body temperature, even after some days (personal communication from Dr. Joshua Carson).

ST degrades lactate due to its composition (Polylactid). The increase in the ionized lactate level signals hypoxic conditions to cells despite normal oxygen levels without changing the actual pH [24]. It serves as an alternative energy source by the pyruvate and lactate transfer [25], enhances angiogenesis, and generates fibroblasts and extracellular matrix [26,27]. Groussard et al., and, recently, Gürünlüoglu et al., demonstrated lactate’s ability to act as a scavenger of free radicals demonstrating the influence on the inflammatory response [9,28]. A positive effect on wound healing was demonstrated compared to Hydrofiber Ag, showing increased keratinocyte generation and faster healing [29,30].

### 3.4. Wound Preparation

#### 3.4.1. Wound Bed Preparation

Preparation of the wounds before applying the epidermal templates can be considered similar in both products. After cleaning, debridement, and necrectomy [12], both products were usually applied under general anesthesia [20,21,31,32] or moderate-to-deep sedation [22], primarily due to the patient’s stress after the injury. The wound bed preparation technique varies among the studies: abrasion was performed using scratchpads or other metallic sponges, brushes, dermabrasion, Versajet, or dermatomes [21,32]. Generally, wound bed preparation was done similarly, depending on the burn depth, and necrosectomy was sometimes performed to induce punctate bleeding [33].

#### 3.4.2. Template Fixation

For template fixation, most authors used staples for mechanical fixation of PX [31,32,34,35,36] and in some cases fibrin glue [20,22] cyanoacrylate glue [20] topical skin adhesives [36], or sutures [21]. Alternatively, xenograft fixation on superficial partial-thickness burns was achieved by 1-day compression [32] and additional dressing changes on day 1 in PX studies to drain blood or serum retention and control the substitutes’ adherence. Frequently, splints were used during the first days to reduce the mobilization of extremities.

However, ST was not mechanically fixated in most studies [10,19,37] with only a secondary dressing holding it in place (see below).

#### 3.4.3. Separation Layer

A separation layer was applied between the product and an absorptive protective dressing in both groups with different dressings, such as antibiotic-loaded agents, silicone, fatty gauze, or nylon dressings being used.

Troy et al. used external dressings with a separation layer until the first dressing change on postoperative day 1, and the PX was exposed to air [32].

### 3.5. Healing Time

#### 3.5.1. Healing Time in Partial Thickness Burns

The comparison was impeded by a missing or inconsistent description of the healing status.

Effects of grafting or conservative treatment were not specified. Therefore, the healing time was considered in uncomplicated wounds without infections or transplantations.

The two treatment groups had a significantly different TBSA with ST mean 11.36 ± 7.37% and PXs with 4.79 ± 5.78 (*p*-value of 0.035) or as weighted data 11.72 ± 7.37 and 1.58 ± 3.44 (*p* < 0.001). Nevertheless, the healing time was not significantly different (*p* = 0.067).

#### 3.5.2. ST Studies

Data were derived from 16 ST studies with 676 patients (See details in Table 2). Eight were excluded as no data at all or no sufficient data on healing time were provided. The remaining nine studies weighted on the number of patients: a mean healing time of 13. 59 days with a mean TBSA of 11.73% ± 7.37% can be reported. The study populations were composed of children, adults, or both. Rashaan et al. found the healing time range’s upper values to be 38 days and 29 days, respectively.

#### 3.5.3. PX Studies

Thirteen PX studies included 1136 patients (see details in Table 3), and seven of them did not provide sufficient data for comparison of healing time and were excluded. After weighting, a mean healing time of 13.22 ± 2.79 days was found in the remaining six studies. The TBSA in studies of patients treated with xenografts had a weighted mean of 1.58 ± 3.44%.

In the xenograft studies, the maximum healing time was 42 days [22]. Duteille reported excision 7.6 days after injury, and healing occurred after 13.4 days in all but three patients.

### 3.6. Change of the Templates or Discontinuation of Treatment

Troy et al. described adhesion loss in their PX studies in 6.8% of patients [32]. Klosova et al., using XE Derma, found adhesion loss in 16% of patients and at least partial disintegration of xenografts in an additional 12% of patients [36]. Out of eight xenograft studies describing unexpected or not defined autografting, adhesion loss was found in five studies, and xenograft change was done between daily and every third day in three studies.

Early detachment or poor wound healing was mentioned in three of the ST^®^ studies. In all these wounds, conservative treatment until wound closure was performed due to the residual defects’ small size. Two studies described at least a partial removal of ST^®^. In one study, early detachment occurred in 33% of the patients [16], attributed to the method of debridement or dressing. In the other study, in three of 15 patients, a dressing removal was necessary without a reason given [14] (Table 4).

### 3.7. Auto-Grafting as Indicator for Burn Wound Conversion

Sufficient data on grafting rates were mentioned in 13 and 17 studies in the PX and ST groups, respectively.

In PX studies, Troy et al. described excision and autografting in 4.5% of patients in a “no variable burn depth group with only partial-thickness burns” [32]. In their retrospective, unselected study, Elmasry et al. [20] had a grafting rate of 30% due to non-closure after two weeks. Details are shown in Table 5. Only clearly defined grafting procedures were included in the table. The time to evaluate the necessity of the use of autografting varied. Blome-Eberwein evaluated skin grafting after three weeks, while Schriek and Sinnig did their evaluation after 11 to 14 days.

According to the studies analyzed, treatment resulted in a mean grafted rate of 2.50% ± 4.05% per ST and 8.63% ± 13.14% per PX study (*p* < 0.0001) as weighted values.

The same effect could be verified by evaluating the statistical effect size of 0.58, demonstrating a medium effect of ST to reduce grafting.

### 3.8. Infection Rates in Partial Thickness Burns

Infection rates were described in 11 studies on PX and 14 on ST. Infection was evaluated only where explicitly described as “infection” (Table 6). Reasons for autografting might overlap these results, as they were not distinguished to prolonged healing time or infection.

#### 3.8.1. Infection Rate ST

Weighted infection rates in the ST studies were 3.83 ± 6.34 in the ST studies. In 24 of 631 (3.8%) participants, a wound infection was described in the ST studies with no difference between pediatric and adult patients.

#### 3.8.2. Infection Rate PX

Weighted infection rates in the studies was 3.83 ± 6.34 in the ST, and 7.04 ± 15.62 in the PX studies. No difference could be found between pediatric and adult patients (*p* = 0.10).

### 3.9. Pain Reduction

Both products were found to reduce pain.

In the ST group, Everett et al. demonstrated a significantly reduced need for intravenous narcotics after ST application [5]. A direct comparison was not possible due to the use of different scales used to investigate pain.

VAS with different ranges were used by Schwarze et al. [19], Blome Eberwein et al. [4], and Hundeshagen et al. [11], showing pain reduction by the ST dressings, partly significant in comparison to other dressings. Wong–Baker and Comfort B scores used by Glat et al. [7] and Rashaan et al. [16] showed values between no pain and minimal pain after ST treatment. Glik et al. [8] showed OASIS superior only on day four without statistical significance.

In the PX group, medication use was evaluated by Burkey et al. [31], finding reduced narcotic doses in 32.4% of the patients and 6.1% needing sedation who did not need it before. Karlsson et al. [22] used Parents Postoperative Pain Measure (PPPM) scores and found no difference in opioid and analgesics use compared to the use of silver foam. Routine use of analgesics was described by Zajicek et al. [45]. Elmasry et al. [20] used the FLACC score, showing a reduction after two days to minimal pain values (3 of 10). Other authors experienced, discussed, or claimed pain reduction without detailed information.

### 3.10. Frequency of the Secondary Dressing Changes

In the study by Fischer et al., the hospital length-of-stay was 69 days, during which nine dressing changes were performed, even though the wounds were closed after 14 days [6]. In five studies, dressing changes were performed every 1–10 days (Table 7).

Often the frequency was described as an interval of dressing changes. Calculating the number of dressing changes, the weighted healing time given in the respective studies was divided by the interval of dressing changes. The number of dressing changes in the ST group was on average 4.38 ± 1.83 dressing changes during the healing period and 4.79 ± 4.29 in the PX studies (*p* = 0.139).

### 3.11. Outpatient Visits and Hospital Length of Stay

Hospital length of stay (LOS) was described in 11 and eight of the PXs and ST^®^ studies, respectively, in different non-comparable modalities. The number of outpatient visits and hospital length of stay depends on the frequency of dressing changes, the burn unit’s policy, and the study design. Burn severity might also influence hospital LOS, which could not be considered due to insufficient data. In prospective ST and PX studies, hospital LOS ranged from 0 [5] to 23.3 days [9] and 2 to approximately 40 days [8], respectively.

### 3.12. Results of the Literature Review on Other Indications for Epidermal Templates in Burns Treatment

When covering freshly harvested keratinocytes after seeding and culturing or precultured keratinocytes, PX did not adhere to the keratinocytes and, therefore, did not survive the first week [46].

In a prospective study of 19 patients, ST was successfully used to cover sprayed keratinocytes in deep dermal burns of the face, with excellent cosmetic outcomes [47]. Moreover, similar results were found in a retrospective study of 103 patients with keratinocytes applied to deep partial-thickness burns and covered with ST [48]. The studies mentioned above showed a mean healing time of 8.04 days, which was shorter than that in the literature wherein other dressings were used [49,50,51]. Neither other wound-associated infections nor patient age influenced the duration of wound healing.

In the sandwich technique, both ST^®^ and PX can be used over a meek graft or a widely meshed autograft to reduce the risk of infection and fluid loss [52].

### 3.13. Results from the Literature on Oxidative Stress during Burns Treatment

Karlsson et al. compared C-reactive protein (CRP) levels during treatment with a silver foam dressing and found lower levels in the PX group without significant intergroup differences [22]. Feng et al. [53] used PX and found a significantly decreased CRP level than in the use of betadine gauze [53]. Iwase et al. could demonstrate that an IL-6 antagonist could reduce the inflammatory response on pig derived transplants, but not on D-dimer [54].

ST decreases total oxidant capacity, increases total antioxidant capacity [29], restores telomere length [9], reduces IL-6 and TNF α activity, and increases TGF-β generation [55] over two weeks in comparison to a silver-containing Hydrofiber product, possibly mediated by the radical scavenging ability of lactate released during degradation accompanied by a shorter healing time [29,55].

## 4. Discussion

PX’s disappearance from the United States market raises several fundamental challenges for burn treatment and the question of the best available replacement.

### 4.1. General Aspects

#### 4.1.1. Viral and Prion Safety

Concerns about the safety of biological products are accompanying the use, at least as a theoretical consideration. In Internet-based research by Wurzer et al. [56] with 111 burn specialists over 36 countries in 2016, the participants rated the risk associated with xenografts as essential in only 32%, which may have changed during the current pandemic situation. The approximately hypothetical risk has been well-known over time [34]; however, epidermal skin replacement’s urgent need supported the application. Unique methods nowadays even might allow for the use of virus-free animals, at least for transplantation trials with pervasive and expensive means so that they are not in general use.

A fully synthetic and biocompatible epidermal skin substitute makes a biological risk assessment needless, as it poses no viral or prion or (probably) even nowadays unknown pathogens risk.

#### 4.1.2. Biocompatibility

Not decellularized PX’s lack of biocompatibility is caused by endothelial membrane-bound Gal and non-Gal antigens. Besides, human monocytes can also recognize porcine endothelial cells [57] causing thrombosis in the template and hindering PX incorporation in the dermal scaffold. The decellularization procedure might reduce thrombosis and increase viral safety to a more theoretical aspect, cross-linking of collagen by aldehyde treatment reduced antigenicity, and rejection and inflammation but could not eliminate it [58,59,60,61]. Even when PX does not vascularize, it remains a biological cover, thereby increasing inflammation as described by Salisbury and Vanstraelen [62,63]. Moreover, the lack of vascularization led to frequent dressing changes in many studies [41], a high rate of unexpected autografting [31,36], prolonged topical wound care after dissolution [36], and the generation of granulation tissue in long term use [21].

Biogenetically reengineered PX could avoid these unwanted effects; nonetheless, it is not yet clinically used [64,65]. Troy et al. [32] discussed rejection and stated a “self-limiting effect by host epidermis reconstitution under the dressing” in partial thickness burns.

The observed, but until now unpublished “xenograft reaction” with leukocytosis and fever might be provoked by this.

Although no actual trans-species viral transmissions are reported in the PX, a potential risk remains [66]. Hume et al. described mitigating factors in viral inactivation such as sample volume and protein content and underscored the necessity to evaluate inactivation protocols of BSL-4 pathogens (viruses) using “worst-case scenarios” [67]. Risks are eliminated with the non-availability of PXs are no more available. Other potential risks of biological replacement products like prions were unknown until the first cases with Creutzfeldt Jacobs Disease remain.

Karlsson et al. compared C-reactive protein (CRP) levels during treatment with a silver foam dressing and found lower levels in the PX group without significant intergroup differences [22]. Feng et al. described a lower CRP level to controls in the early and late treatment phases and hypothesized a positive effect on SIRS by PXs [68] but Iwase et al. demonstrated evidence of a sustained systemic inflammatory response [54]. 

ST^®^ is biocompatible, fully resorbed without a foreign body reaction, and does not cause rejection as tested in CE and FDA 510 k clearance. Shelf-life discussions are irrelevant in a non-available product. Other similar products are not the topic of this paper.

#### 4.1.3. Ethical and Religious Considerations for a Replacement Decision

Non-availability of PXs eliminates, at least in the US, Deliberations linked to the use.

In the areas of the world with pigskin production like XE-Derma [45], the aspects as described by Eriksson et al. [69] are still relevant: Sunni and Shiite Muslims who reject porcine-derived products, whereas, for Hindus and Sikhs, these are acceptable if no alternative product is available and if the treatment is considered life-prolonging. In Iran, lyophilized PX has been legalized [21]. Therefore, PX use requires the patient’s informed consent or its legal deputy [70]. For ST^®^, no ethical, cultural, or religious limitations are described as a fully synthetic product.

### 4.2. Usability

#### 4.2.1. The Usability in Donor Areas

The safe and effective treatment of donor areas is of concern, as these artificially created wounds are of partial thickness, and nonhealing donor areas may prolong morbidity.

The use in donor areas was seen differently. Although PX is described as indicated for donor site closure, many authors disagreed with this because it might trigger local site inflammation [22,62,63,71]. ST^®^ is widely used to cover donor sites [7,72,73,74], and many authors described a positive impact on wound healing, pain control, patient comfort, and ease of use [5,7,72,73,75,76,77].

#### 4.2.2. Covering Keratinocytes

When used as a cover for cultured keratinocytes, PX did not adhere to the wounds, and the keratinocytes did not survive the first week [46] no matter whether precultured or not-precultured keratinocytes were used. In a prospective study of 19 patients, ST was successfully used to cover sprayed keratinocytes in deep dermal burns of the face, with reasonable cosmetic outcomes [47]. Moreover, similar results were found in a retrospective study of 103 patients with keratinocytes applied to deep partial-thickness burns and covered with ST [48]. The studies’ results revealed a mean healing time of 7.34 ± 2.84 days after application, which was shorter than that in the literature wherein other dressings were used [49,50,51]. Neither wound-associated infections nor patient age influenced the duration of wound healing in this case-series.

#### 4.2.3. The Use as a Sandwich Technique

Using a sandwich technique, both PX and ST^®^ have been used successfully over Meek grafts or widely meshed autograft to reduce the risk of infection and fluid loss [52,78]. The potent pain-reducing abilities of ST^®^ and the reduced number of dressing changes may be advantageous in this indication.

#### 4.2.4. The Use for Preparation of the Wound Bed by Xenografts

Xenografts can be used to prepare the wound bed before grafting, thereby creating granulation tissue in deeper parts [21], and ST can be used to prepare the wound bed as well [79] and to induce tissue neoformation and is reported to reduce the sizes of areas to be grafted and therefore donor areas [37].

### 4.3. The Use of the Products to Provide Undisturbed Wound Healing

Healing time, the frequency of dressing changes, the rate of infections, dissolution of the epidermal skin substitute, grafting rates, and pain during treatment and dressing changes might be indicators for undisturbedness.

#### 4.3.1. Healing Time

Data are presented in Table 1 and Table 2. Healing time only seems to be an easy parameter for undisturbed wound healing. The number of dressing changes, infection rates, and grafting rates is other parameters. The healing time evaluated in this paper was the time of uncomplicated healing in wounds without transplantations. When evaluating healing time, the number of patients grafted has to be considered, as must be considered, as the indication for grafting might be a predictable prolonged healing time. It also has to be considered that the wounds covered with ST were nearly six times as large as those covered with PXs.

##### Healing Time in Partial Thickness Burns

With similar inclusion and exclusion criteria, the healing time in uncomplicated wound healing was in the ST Ø 13.59 ± 1.86 days and the PX group Ø 13.22 ± 2.1 days after weighting the data.

Comparison of weighted data showed a healing time in the ST studies, with a statistically not significant difference of *p* = 0.067. The difference might influence this in weighted TBSA, which was about seven times as high in the ST group (11.36 ± 7.37%, compared to 1.58 ± 344%), a significantly higher infection rate (3.85 ± 6.35 versus 7.03 ± 15.65). Early grafting based on the evaluation that no spontaneous healing was expected within three weeks and early infections may have classified patients as drop-out for wound healing time evaluation and shortened by this the PX average healing time. The impact on the standardized effect size of mean wound healing days was small (0.19).

No study provided data with a healing time without infections and grafting as signs of undisturbed healing in the xenograft group.

In the ST group, undisturbed wound healing was reported in six studies with 218 patients.

In the ST^®^ studies, 96.8% of the patients healed without transplants, while 91.7% in the PX studies. Infections without transplantation prolonged the healing time from about ten days to 16 days; the healing time after transplantations remains unclear.

##### Mixed and Deep Partial Thickness Burns

The treatment of mixed and deep partial-thickness burns is of high interest, as the standard procedure suggested for this condition is grafting [37]; treatment with an epidermal skin substitute may reduce the area grafted, thereby reducing donor sites. Grafting in partial-thickness burns has cosmetic consequences, especially with mesh grafts [37], where a graft pattern and graft margins may remain visible. Healing time^®^ in mixed burns is an essential parameter for the choice of conservative or operative treatment and ranged from 8.4 [9] to >38 days, indicating the presence of minor full thickness burns or the influence of infections on the healing process.

Healing time in mixed burns in the xenograft group was described by Bukovcan et al., who reported a correlation with TBSA. Patients with a TBSA < 10% and >20% had healing times of 13.6 ± 11.1 days and 24.6 ± 12.7 days, respectively. The mean healing time not regarding TBSA was 13.47 days in PX treated children and in adults, the mean healing time was 15 days in their study. Highton et al. [10] described a median healing time in their superficial and deep dermal wounds of 16 days.

Therefore, no conclusions can be drawn. When looking at the results, most studies with xenografts only described healing in parts of the patients after thirty days.

Other components like clinical practice might influence the results: Elmasry had a grafting rate of 30%. Nevertheless, in TBSA and burn depth analysis, superficial second-degree burns in his study had a mean TBSA of 5%, and deep second- and third-degree burns only had a TBSA range from 0 to 0.1%, so the depth of wounds could not be the reason for the higher grafting rate.

The healing time in deep partial-thickness burns with completed healing within 30 days as demonstrated by Keck et al. with ST^®^ compared to that of PX, as reported by Hosseini et al. [21] revealed that after one week, stage four granulation tissue was found in 13% of the PX patients (see Table 7). The results are lacking statistical validity.

#### 4.3.2. Burn Wound Progression

In some studies, wounds were covered in mixed and deep burns until definitive healing or grafting [4,18,37,80]. As shown in longitudinal and comparative ST^®^ studies, a temporary covering predisposes to partial spontaneous healing and limits the areas that must be grafted.

ST^®^ is possibly causing less irritation and positive healing effects [29,55]. Both ST and PXs trigger faster epithelialization than does silver sulfadiazine and povidone-iodine cream [21,53]. Healey et al. described no significant difference in healing time between PX and paraffin gauze [40]. The reduced grafting rate in ST studies might indicate a reduction of burn wound conversion.

The reduction of oxidative stress is an essential prerequisite in ongoing wound healing. Dressings can have systemic effects, as demonstrated by occlusive dressings [81]. Karlsson et al. found lower CRP levels, indicating reduced oxidative stress when comparing PX efficacy with that of silver foam in partial-thickness burns; however, PX will trigger an immune response in wounds.

Ogawa found chronic inflammation as an essential trigger of hypertrophic scarring [82]. Gürünlüoglu et al. demonstrated that polylactide epidermal substitutes exert positive systemic effects on oxidative stress in burns’ pathophysiology [29,30,55]. These positive effects were explained with a new understanding of lactate’s role in energy distribution, utilization, and radical scavenging. The rate of hypertrophic scarring was not investigated in a direct comparison of PXs, and therefore only personal impressions about a better scar outcome in ST^®^ treated are reported [4,29,83].

#### 4.3.3. Temporary Cover of Full Thickness Burns

Both products have been used for the temporary closure of full-thickness burns. Middelkoop, Grigg et al., and others described the use of PX for this indication [80,84]. However, they provide no information about the maximum duration of the temporary closure. Heimbach et al. described PX use as limited to 7 days due to a reduced resistance against infection [85,86]. Saffle concluded that PX was less effective than allograft in excised burn wounds [87]. 

Chiu et al. did not include full-thickness burns as an indication for PX in their review [34]; nevertheless, it is used with frequent material changes. Notwithstanding, a previous study reported partial healing of full thickness wounds in very young pigs after applying freshly harvested PX only [88].

Small full-thickness areas can be covered with ST^®^ until complete wound healing [75]. Case reports describe the temporary closure of excised burn wounds for up to 3 weeks [89,90] under the same surgical conditions as temporary dermal templates. So far, ST^®^ has been used as a temporization product, although with insufficient evidence.

#### 4.3.4. Use as a Dermal Template in Supporting Tissue Replacement and to Bridge Time to Availability of Donor Skin or CEA

In deep dermal burns, where there is limited availability of donor areas, mono- and bilayer dermal regeneration templates [91,92,93] of biological or biosynthetic or fully synthetic origin [94] can help bridge the time until skin grafts or cultured epithelial autografts or dermal–epidermal substitutes [8,95,96,97,98] are available again. Other methods use pathogen-free human keratinocyte progenitor cells to replace autologous epidermal cells [99] and can be used immediately, as demonstrated in traumatic wounds [100].

Dermal templates can help to improve the stability of the new dermo-epidermal constructs and the cosmetical outcome [92]. The use of Suprathel as a dermal template or in covering full thickness wounds temporarily has been demonstrated in single cases but not described in studies [89,101].

Polylactic membranes might even have a positive effect on osteogenicity [102] and might be helpful to support techniques like the “induced membrane technique” for replacement of bone loss [103] or in maxillofacial surgery, porcine bone xenografts were tested in a non-inferiority study to bovine-derived xenografts in rat calvaria with good results.

### 4.4. Pain Reduction

Reduced pain and workload are essential features during wound healing and enable early mobilization and early weaning from the ventilator with reduced stress for patients and staff. Pain reduction might even help to reduce opioid dependency after burns treatment. Both products were shown to reduce pain [7,31,41,73]. The only direct study comparing ST^®^ and PX efficacies on pain control was conducted on TENS and not on burns. Lindford [104], in a case report, found no pain in the ST^®^- and xenograft treated areas; however, the allograft-treated areas were painful during movement.

In the xenograft studies, Burkey et al. [31] evaluated the effect of PX on pain using the need for intravenous narcotics and moderate sedation in each patient. They found less use of intravenous narcotics in 32%, unchanged in 61%, and increased by 6.7%. Therefore, positive effects on pain could be seen in 32% and no or adverse effects in the rest. The sedation reduction effect was more pronounced, as only 35% did not show a positive effect. Sixty-four percent of patients no longer received sedation. In 29.9% of patients, no change in use was found, and 6.1% of patients who did not receive preoperative sedation received it postoperatively.

Elmasry found a significant reduction in the Face, Legs, Activity, Cry, and Consolability (FLACC) scores, initially ranging from 3 to 7 and decreased after day 3 to <3, which could be interpreted as mild discomfort [20]. Karlsson et al. found no difference in pain at any time when comparing the efficacies of xenografts and silver foam [22]. However, the dressing was applied with Safetac, which might reduce pain by itself [105]. Dressing changes were conducted under ketamine and midazolam, propofol and fentanyl, and, in some cases, even under sevoflurane [22]. Zajicek needed analgesics in 90% of his pediatric patients and 100% of his adult patients during the first seven days of dressing changes [45]. Bukovcan et al. [38], Hobby et al. [106], Priebe et al. [41], and Troy et al. [32] found a positive effect on pain reduction.

In the ST^®^ group, Everet et al. [5] reported delivery of intravenous narcotic doses with 1.5 before ST^®^ and 0.1 shortly after ST^®^ application. The average pain score at the first follow-up visit was 1.2/10, comparable to Blome-Eberwein et al., who reported an average pain scale score of 1.9/10, both without describing variance interpreted as a moderate pain the study in partial-thickness burns over the entire period [4]. Glat et al. [7] used the Wong–Baker face pain scale score and calculated a pain score of 1.2/10 shortly after debridement and ST^®^ application. Schwarze et al. [72] reported a median pain VAS score of 0.9/10, compared to that using Omiderm of 1.59. Hundeshagen et al. [11] showed a significant reduction in pain during the first 20 days compared to Mepilex Ag^®^, especially in children. Rashaan et al. [16], using Comfort B scores, described only minimal background pain and procedural pain changes. Fischer et al. [6] reported positive side effects: the avoidance of secondary pain killers and sedative drugs during dressing changes contributed to stability. Only Glik et al. [8] found inferiority in pain reduction measured by VAS on day 5 with ST^®^ than with Oasis, without statistical significance; however, all studies comparing pain reduction seemed to show a more substantial ST^®^ effect, where no statistical comparisons could be made.

### 4.5. Infection Rates

Infections are serious adverse effects in burns treatment. Infections, premature detachment, wound colonization, and possibly unexpected grafting are critical irritations in wound healing, which are only partially described. Infection rates seemed to be higher in deeper wounds, extensive burns, and burns treated later after injury.

Infections and the number of early dissolutions of ST^®^ and PX might be reflected in the number of external dressing changes. Infections prolonged the healing time with ST^®^.

In weighted cases, a statistical difference between the treatment groups could be identified with a *p*-value of <0.001. Nevertheless, efficiency measured by Cohen’s d only showed a small effect on infection reduction of ST compared to PXs.

A higher infection rate indicated deeper burns or necrotic tissue persistence. Closure with an epidermal template might influence the infection rate. Iqbal et al., who initially washed and debrided the wound from dead tissue in superficial, mid-dermal, and deep dermal burns, had 20 patients (31%) with healing >21 days and a strong association of longer healing time with infections. Similarly, Rashaan et al. found that only patients with wound infection had prolonged wound healing.

Xenografts are described as limiting bacterial growth [52,107], whereas ST^®^ forms a bacterial tight barrier [108]. Karlsson found no differences in C-reactive protein or core temperature between PX and silver foam use [22] as indicators for reduced inflammatory response. ST^®^ has the feature of bacterial impermeability and reducing systemic oxidative stress compared to a silver product [29].

### 4.6. Grafting Rates in Partial Thickness Burns

One of the indications of skin substitutes in burns is the intention to reduce burn wound conversion. Some have different definitions of burn wound conversion; therefore, it is a pragmatic approach to evaluating the unexpected grafting rate in partial thickness burns after a specific time. Grafting should generally be performed within three weeks in order to avoid hypertrophic scarring [109].

The studies’ different grafting frequency demonstrates varying evaluation modalities of the grafting necessity and reflects different patient inclusion criteria and different ways of classifying partial-thickness burns. Wounds not entirely healed with minimal residual defects after detachment of ST^®^ or PX were treated conservatively in both groups until healing was attained.

In PX studies, Burkey et al. [31] (superficial partial-thickness as inclusion criterion) reported that 14% of patients needed unexpected autografting, Duteille et al., (undetermined face burns as inclusion criterion) reported this in 3/20 patients [35], Elmasry et al. [20] (superficial and deep partial-thickness as inclusion criterion) needed an operation in 20% of patients. However, his study contained nearly no full thickness burns. Klosova et al. [36] (partial-thickness as inclusion criterion) reported early dissolution in 19% of patients. Troy et al. [32] (charge codes as inclusion criteria) reported premature graft separation in 6.8% of patients.

Grafting after application in the ST^®^ studies in partial-thickness burns was 0% in the Everett et al. study (*n* = 17); Blome-Eberwein et al. (*n* = 227) found no areas to be grafted, 2.4% were treated topically due to minimal size of residual defects. Patients in the Hundeshagen et al. study (*n* = 31), in 3%, needed grafting; Schwarze et al., (*n* = 30) excluded patients with Abbreviated Burn Severity Index >10 and showed a skin grafting rate of 0%. Rashaan et al., (*n* = 21) found problems with ST^®^ adherence attributed to insufficient debridement with a grafting rate of 14%.

The average grafting rates derived from single studies were 2.5 ± 4.06 and 8.63 ± 13.14 demonstrating the difference, supporting the calculated efficiency of 0.52 with a *p*-value < 0. 001 and a power of 0.99.

### 4.7. The Frequency of Outer Dressing Changes

The frequency of outer dressing changes might be a summative effect of undisturbed wound healing, as it reflects infections, unexpected dissolution of the epidermal skin substitute, and unwanted effects derived from dressings, and the number of controls estimated as necessary. It also reflects the workload for the staff.

It was calculated as the number of dressing changes until the wounds were healed. On average, the ST^®^ treated patients had 4.38 ± 1.83 dressing changes, and the PX treated patients 4.79 ± 4.28. However, the difference is not significant (*p* = 0.139 Wilcoxon Test). As the data might be derived on study schedules, this limits the meaning. Nevertheless, the difference might mean fewer unwanted situations and a lower workload in the ST^®^ group.

Elmasry et al. performed daily dressing changes [20]; this frequency seemed predetermined by the study protocol. In the study by Karlsson et al. [22], up to three outpatient visits and external dressing controls were performed weekly. Troy et al. [32] performed weekly wound surveillance. Duteille et al. [35] scheduled follow-up visits on day 14 after the facial treatment. Hosseini et al. reported a mean hospital LOS after PX of 4.69 days and a mean number of dressing changes of 1.5 after PX application. Patients were discharged after ST^®^ Treatment the same day or the next day by Glat et al. [7].

### 4.8. Hospital LOS

Depending on the burn severity, the procedures applied in the different burn units, and complications, and the number of outpatients visits heretofore may reflect the study protocol. The average patient hospital LOS ranged from one day to 16 days in the PX studies and 0 to 23 days. Two studies were excluded from this report: an 81-year-old patient with a 51% TBSA burn and 55 days LOS [8] and a 40 days average in a comparison study with OASIS in the ST^®^ [39] studies. It has to be considered that LOS can be reduced substantially when the outpatient treatment infrastructure is adapted to the needs.

### 4.9. Use of Both Product Categories in Other Fields of Trauma

In other indications as mechanical trauma, partial thickness wounds, donor areas for skin grafting, and temporary cover of skin defects might indicate both products. To reduce the consequences of surgical trauma, Suprathel also was used successfully as a peritoneal adhesion barrier in abdominal surgery [110] and as a pericardial adhesion barrier in cardiac surgery [111].

Many other products are in use for superficial and partial thickness burns and donor sites, but a comparison to Suprathel was not the paper’s topic.

## 5. Conclusions

ST has a broad range of indications and has become the dressing of choice in many burn centers to treat partial thickness burns and donor areas, and it can be used successfully to cover sprayed keratinocytes. It appears to enable undisturbed wound healing at a substantially higher rate than PX. With an equal healing time, fewer infections, and a significantly lower transplantation rate, a lower number of dressing changes that were not statistically significant and may be based on study protocols during treatment of partial thickness burns supports wound healing even in more extensive burns. It reduces burn wound progression better than PX. Although no direct comparison was possible, there are strong indicators of more significant pain reduction and increased treatment comfort for patients and the team under ST treatment, as visible in the comparison of effectiveness data.

Although limitations exist regarding comparability, ST^®^ treatment appears to be the right choice for PX replacement in the above-outlined indications. The fully synthetic and biocompatible off-the-shelf product is safe and cannot transmit viral or bacterial diseases, unlike other biological products. We hope to evaluate the ongoing results as ST^®^ entirely moves to replace PXs. We suspect ST^®^ will be superior to PXs, but this will need to be rigorously studied.

## 6. Limitations

In nearly all the studies, the diagnosis of partial thickness burn was solely based on clinical assessment. No study has objectively evaluated burn depth, for example, by laser Doppler imaging. Therefore, the differentiation of superficial partial-thickness and deep partial-thickness burns or partial full-thickness burns remains somewhat questionable. Many PX studies were retrospective investigations based on current procedural codes; thus, the primary indications may have differed.

The studies were based on an average TBSA in the groups, which were approximately only one-sixth of the ST studies in the PX studies.

A definitive treatment intention or a diagnostic evaluation of wound healing potential might have been the indication for PX use; however, this was not defined in the studies. The same applies to some ST^®^ studies, where the progress of wound healing up to a specific day was observed to minimize the grafted area. The low rate of PX studies with a definitive time of healing reduced the comparability and the incompleteness of the description. The study misses result on parameters, as pliability of the skin and functional impairment, and a long-time outcome that was not described sufficiently and in the numbers to be comparable.

This comparison was based on partial thickness burns and wounds, as ST^®^ was mainly used for this purpose. In a few cases, however, ST^®^ was placed on small full-thickness areas. Although some centers have successfully used ST^®^ to temporize excised full-thickness burns, there are no studies on this topic. Therefore, this review’s level of evidence is reduced by the small number of studies and non-standardized methods.

To date, there is no side-by-side comparison of ST to Xenograft, and likely will not be one given one as PX is no longer available. Nonetheless, this manuscript describes the advantages of utilizing a safe, allogenic alternative for burn care as PX’s old technology phases out. Data quality limited the statistical evaluation, and the results should be seen with caution.

## Figures and Tables

**Table 1:** Calculation of the effect size for healing time, percentage of infections, the percentage to be grafted, and dressing changes with weighted data. Stdev = standard deviation.

	Healing Time	Infected %	Grafted %	Dressing Changes
Mean ST	13.59	3.83	2.50	4.38
Mean PX	7.03	7.04	8.36	4.79
Stdev ST	1.86	6.44	4.05	1.83
Stdev PX	2.09	15.62	13.14	4.28
Sample size ST	371	625	681	398
Sample size PX	143	1124	1136	286
Standardized Effectsize at 95% Confidence Intervall	0.19	0.2	0.52	0.13

**Table 2:** Healing time in ST studies.

Name of the First Study Author	Number of Patients	Study Design	Age	TBSA	Inclusion	Healing Time Days
Blome Eberwein [4]	229	Retro	P (Pediatric): 138, a (adults): 91	Ø 8.6 1–60.5	Superficial and deep second degree	Mean 13.7 d (days) p: 11.9 d A: 14.7 d
Everett [5]	17	Retro	P, Ø 33 m (months)	Ø 5%	Superficial and partial thickness	Mean 9.4 (5–24) d
Fischer [6]	1	Case report	A, 81 a	51%	Partial thickness	14 d
Glat [7]	12	Prospective	Ø 3.6 y (years)	Ø 5.5%	Superficial and mixed	Mean 8.4 d
Glik [8]	24	Retrospective unblinded pair control	Ø 48 y (21–86 y)	Ø 23.8	Burns of both hands to minimize differences	From Figure 1: complete healing d 20
Gürünlüoglu [9]	20	Prospective randomized	4.9 ± 3.8 y	Ø 31.95 ± 4.43%	Acute burns, 1–60 y, 20–50% including deep burns 5–10%	Median 13.5 d (range 9–21 d) Mean 14.25 ±3.46 d
Highton [10]	33	Prospective	P: Ø 29 m (5 m–11 y)	Ø 4 (1–13)%	Superficial partial *n* = 24 mid-dermal: *n* = 19 deep *n* = 10, >21 d and infection	Median 16 (range 9–38) d; Mean 19.5 ± 8.4 d
Hundeshagen [11]	30	Prospective randomized	A: Ø 24.0 ± 23.0	Ø 5.5 ± 4.6%	Partial thickness, FT excl.	Median 12.0 d,
Iqbal [12]	65	Prospective	Ø 4.9 y (4 m–11 y)	Ø 23.6% (8–45)	Superficial dermal 16, mid-dermal 34, deep-dermal 15	Mean 15 (10–35) d
Kukko [13]	8	Retrospective	Ø 18 mo, range 10–39	Ø 7.6 Stdev. missing	Scald injuries	All burns healed by the end of the third week.
Madry [14]	15	Retrospective	1 p, 14 a	Not defined	partial thickness within 96 h after injury	Application: (a) ≤24 hs; (b) 24–48 h; (c) >48 h
Radu [15]	30	Prospective randomized	Median 42 y, (range 18–80 years)	Ø 18% (range 6–36)	Superficial partial thickness burn >3%	Not defined
Rashaan [16]	21	Prospective observational	Median 2.4 y (range 5 m–14 y)	4.0% (range 1–18)	All consecutive partial thickness burns < 48 h after injury and age < 18 years SPTB: 12 DPTB: 9	Median 13 (range 7–29); without bacterial contamination: 13 (7–18); with bacterial contamination 15 (9–29) Mean: 15.5 ± 6.36 d
Schiefer [17]	24	Prospective randomized	Ø 39.8 ± 18 y	0.5 ± 3.0%	All patients with superficial partial thickness burn of the hands	All patients after 7 to 10 days healed completely
Schriek [18]	149 (last year)	Retrospective	Pediatric	Not defined	All partial thickness burns	After 10–12 days, 7–9% grafted
Schwarze [19]	30	Prospective, randomized bicentric	a	1.5%		0

**Table 3:** Healing time in PX studies.

The Name of the First Study Author	Number of Patients	Study Design	Age	TBSA	Inclusion	Healing Time
Bukovcan [38]	109	Retrospective	Ø 7.6 ± 15.3	Ø 13 ± 8.2%	Superficial and partial thickness burns	Ø 15.1 d ± 11.6 total
Burkey [31]	164	Retrospective	Pediatric	Ø 5.8 ± 4.4%	Superficial partial thickness burns	Not described
Chiu [34]	2	Case reports	Ø 14	Not described	Partial thickness burns of the face, mesh graft pattern	Healed after 10 days
Diegidio [39]	534	Retrospective	Ø 3.41	Ø 8.41%	Scalds from ABA and own registry	Not described
Duteille [35]	20	Prospective	Ø 16.45% range	Ø 27.75%	Intermediary 2nd-degree facial burns	Initial healing time after excision: Ø 13.4 d, 3 grafted
Elmasry [20]	67	Retrospective	Median: 1 y, IQR 1–2	Median 6.2IQR 4–11	Scalds treated with xenograft (deep and FT)	Not defined
Healy [40]	16	Prospective randomized	Ø 2.6 y ± 7.0	Ø 1.8 ± 0.8%	Partial- thickness burns < 10% BSA	12.9 days in spontaneously healed patients (=47%)
Karlsson [22]	58	Prospective randomized	Ø 21 m (11–59)	Median 5% (3–22)	Partial thickness, <72 h after injury, 6 m–6 y	Median 97% healing 15 d (range 9–29) Ø 17 Median 100% healing: 20.5 range 11–42
Klosova [36]	91	Retrospective	2.5	1–20%	Partial thickness and burn center admission	12–14 d
Klosova	10	Retrospective	42	1–20%	Partial thickness and burn center admission	
Priebe [41]	17	Prospective	15 < 28 m	Not defined	Areas with comparable aspects of 2nd degree	13 of 17 healed in 15 days,
Rodriguez Ferreyra [42]	20	Not defined	Ø 19.2 y	Ø 14.8, no std	Not described	No healing time described.
Troy [32]	133	Retrospective	Ø 17.7, range	Ø 16 ± 37.7%	partial thickness burns, no hands, no pediatric pat	Not described

**Table 4:** Change or discontinuation of Suprathel or PX treatment.

ST^®^			Xenograft		
First Author	ST^®^	Comment	First Author	Xenografts Change or Diss.	Comment
Blome Eberwein [4]	No change and no autografts.	In 5.2% failure or progression to full thickness, residual defects treated conservatively	Burkey [31]	11% of 164 not anticipated autografting + prolonged topical wound care in 6 pat. (3.7%) not anticipated and 22 (14%) anticipated	14.7% (in a total of not anticipated autografting or prolonged wound care)
Everett [5]	No change		Burleson [43] cited by Chiu [34]	Change every two days	Partial-thickness porcine split skin
Fischer [6]	No change		Duteille [35]	EZ derm in place after surgery for three days, followed by grafting or topical wound care	Grafting in 3 patients,
Gürünüloglu [9]	No change		Elmasry [20]	20% needed an operation	No use in hands
			Klosova [36]	19% (81% no signs of dissolution)	XE derma
Hundeshagen [11]	No change		Priebe [41]	EZ Derm replaced every third day	
Madry [14]	No change	One dressing removal necessary when ST applied at 24–48 h; 2 removals necessary, applied >48 h after injury (reasons nor specified).	Rappaport [44] cited by Chiu [34]	Daily change of xenograft	Deep Frozen pigskin
Rashaan [16]	No change, early detachment in 43% treated conservatively	33% contamination before ST^®^, detachment is linked to the method of debridement and topical wound care when detached.	Troy [32]	6.8% with premature graft separation, 15% lost for follow-up	After separation, local wound care
Schiefer [17]	No change				
Schwarze [19]	No change				

**Table 5:** Grafting rates in partial thickness burns.

ST^®^					PX				
Study	*n*=	% Grafted	*n*=	Type of Burn	Study	*n*=	% Grafted	Number of Grafted	Type of Burn
Blome Eberwein [4]	229	0%	0	2nd degree burns superficial and partial	Bukovcan [38]	109	3.7%	4	superficial partial scald burns
Everett [5]	17	0%	0	Partial thickness within 6 h	Burkey [31]	167	5.5% unexpected	3 + 21	Superficial partial-thickness inclusion
Fischer [6]	1	0%	0	Partial thickness	Duteille [35]	20	15%	3	Intermediate face burns
Gürünlüoglu [9]	20	0%	0	Superficial and deep partial thickness burns	Elmasry [20]	67	30%	20	Only superficial partial-thickness burns
Hundeshagen [11]	30	6.6%	1	Partial thickness burns	Healy [40]	32	7 out of 16 EZ Derm 44%	7	Partial, no hands or faces
Iqbal [12]	65	0%	0	Partial-thickness burns	Karlsson [22]	29	13%	6	No palms, soles, or faces
Madry [14]	15	26%	2	Children, Flame and scald burns	Klosova [36]	91 children	30%	27	Partial thickness burns and full thickness
Rashaan [16]	21	14%	3	Superficial. and deep partial, 7% of all patients colonization before ST^®^	Klosova [36]	10 adults	90%	9	Partial thickness burns and full thickness
Schulz [17]	24	0%	0	Partial thickness	Priebe [41]	15	13%	2	Scald burns, children
Schriek and Sinnig [18]	149	9%	11 last year of table	Superficial and partial deep burns	Rodriguez Ferreyra [42]	20	0%	0	superficial
Schwarze [19]	30	0%		Superficial or mid dermal burns	Troy [32]	157	8.6%		6.8 + 4.5 + 2.2 Partial, no hands, no faces

**Table 6:** Infection rates in partial thickness burn studies (p = pediatric, a = adult).

ST					Xenograft				
First Author	*N*=	Infections	Infect. %	Healing Time	First Author	*N*=	Infections	Infect. %	Healing Time
Blome Eberwein [4]	138 p 91 a	0 8	0 8.8%	13.9 14.70	Bukovcan [38]	109 p	4	4%	15.10
Everett [5]	17 p	0	0	9.40	Burkey [31]	167 p	4	2%	insuff. Data
Glat [7]	12 p	0	0	8.40	Diegidio [39]	534 p	3	0.01%	Insuff. Data
Hundeshagen [11]	31 a	1	6.45%		Duteille [35]	20 a	3	15%	insuff. Data
Iqbal [12]	65 p	13	20%	15.00	Elmasry [20]	20 p	7	35%	insuff. Data
Rashaan [16]	21 p	1	4.76%	15.56	Healy [40]	16 a	7	43%	insuff. Data
Schwarze [19]	30 a	0	0	10.20	Karlsson [22]	58 p	9	16%	17.00
					Klosova [36]	101 p + a	5	5%	Nd
					Priebe [41]	15 p	Nd (Not defined)		Nd
					Rodriguez Ferreyra [42]	20 p + a	0	0%	insuff. Data
					Troy [32]	15 a	2	13%	insuff. Data
Average per study		3.83%	±6.34		Average per studies		7.039	15.62	

**Table 7:** The frequency of outer dressing changes.

ST Studies, First Author	Outer Dc Every Day	Approx. Healing Time	Total Number of DC	PX Studies, First Author	Outer Dc Every Day	Approx. Healing Time	Total Number of DC
Blome Eberwein [4]	1–4 (2.5)	14.2	5.68	Burkey [31]	Average DC 1.6	Healing time not described	1.6
Everett [5]	5–7 (6)	9.5	1.59	Bukovcan [38]	2	15.1	7.6
Hundeshagen [11]	3–5 (4)	12	3	Elmasry [20] *	1	12.2	12
Iqbal [12]	4–5 (4.5)	15	3.33	Duteille [35]	3 days then moistened gauze	3 *	* excluded
Rashaan [16]	3	15	5	Karlsson [22]	3 regularly, up to three times a week, Number of DC: 5 (−9), time for DC 20 min (10–50)	Time to 95% healing 15 days	5
				Priebe [41]	3	15	5
The average number of dressing changes during Healing time and		13.61	3.43 ± 1.46 Median 3.165 Range 4.09			14.33	7.4 ± 2.86 Median 5 Range 10.4

* The study of Duteille et al. was excluded, as no exact healing time and dressing changes were provided.

## Data Availability

Data are publicly available, as cited in the references.

## Abbreviations

ST:	Suprathel^®^
PX:	Porcine xenograft
TNF-α:	Tumor Necrosis Factor Alpha
TEN:	Toxic Epidermal Necrolysis
LOS:	Length of stay
VAS:	Visual Analogue Scale
PPPM:	Parents Postoperative Pain Measurement
ns:	not significant
